# Early start and stop of biologics: has the time come?

**DOI:** 10.1186/1741-7015-12-25

**Published:** 2014-02-06

**Authors:** Ronald F van Vollenhoven, György Nagy, Paul P Tak

**Affiliations:** 1 Unit for Clinical Therapy Research, Inflammatory Diseases (ClinTRID), The Karolinska Institute, Stockholm 17176, Sweden; 2 Department of Genetics, Cell, and Immunobiology, Faculty of Medicine, Semmelweis University, Budapest 1089, Hungary; 3 Department of Rheumatology, Semmelweis University, Medical School, Budapest 1023, Hungary; 4 Academic Medical Center/University of Amsterdam, Amsterdam, The Netherlands; 5 University of Cambridge, Cambridge, UK; 6 Current address: GlaxoSmithKline, Stevenage, UK

**Keywords:** Biologics, Rheumatoid arthritis, Treatment

## Abstract

Despite considerable advances in the management of rheumatoid arthritis, results are still not satisfactory for all patients. The treatment goal in rheumatoid arthritis is remission, and there currently are numerous conventional and biological medications available to reach this aim. There are also different treatment strategies but with only limited comparative evidence about their efficacies. More patients now achieve remission while on treatment, but it remains elusive in the majority of patients. Treatment-free remission, the ultimate goal of therapy, is only achieved in very few patients; even when this happens, it is most likely due to the natural course of the disease rather than to any specific therapies. Modern treatment is based on the initiation of aggressive therapy as soon as the diagnosis is established, and on modifying or intensifying therapy guided by frequent assessment of disease activity. In this commentary we will discuss the current treatment paradigm as well as the possibility of an induction-maintenance regimen with biological disease-modifying antirheumatic drugs in early rheumatoid arthritis.

## Background

The goals of treatment of rheumatoid arthritis (RA) include induction of disease remission and protection against progressive joint destruction [1]. As early RA already represents chronic synovial inflammation [2], which may lead to erosive disease during the first months to years after clinical onset of the disease [3], effective antirheumatic treatment needs to be initiated as soon as the diagnosis has been established. It is generally accepted that early control of inflammation results in a better outcome in terms of joint damage, functional status and quality of life. Currently, patients with RA usually start with conventional disease-modifying antirheumatic drugs (DMARDs) and switch to biological treatment if there is persistent disease activity in spite of optimal conventional treatment [1,4]. Whether biologicals might prevent the initiation of RA in high-risk individuals (for example, persons with anti-cyclic citrullinated peptide positivity, arthralgia and genetic susceptibility) is unclear; ongoing studies are attempting to address this question (Tak, personal communication).

Following the advent of anti-TNF therapies, studies with such agents clearly demonstrated that a combination of anti-TNF with methotrexate (MTX) in early RA achieved significantly better results, at least at the group level, than MTX monotherapy [5-7]. A direct comparison between conventional DMARD combinations with MTX plus anti-TNF was needed, and such a comparison was made in both the Swedish Farmacotherapy (Swefot) and the Treatment of Early Aggressive Rheumatoid Arthritis (TEAR) clinical trials. In the Swefot trial, a significant clinical advantage for anti-TNF was demonstrated after the first [8], but not after the second year - the latter finding being attributable both to a slow incremental benefit of the conventional agents and a lack of statistical power (type II error) [9]. The two-year results did confirm that the anti-TNF combination was superior in preventing radiographic damage. The TEAR trial confirmed neither the clinical nor the radiographic benefit of anti-TNF over conventional combination therapy, but featured a more complex design and analysis that may have underestimated the differences between the treatment arms [10]. The NEO-RACo trial was designed to study the long-term outcomes of the combination of MTX, sulfasalazine, hydroxychloroquine and prednisolone treatment with and without additional infliximab therapy [11]. This study showed that the long-term efficacy of the combination of conventional DMARDs and prednisolone was not further improved by adding infliximab during the first six months of treatment.

That disease control can be achieved with conventional DMARDs in a significant proportion of patients, in particular when these drugs are used in combination, is also supported by other studies. The Finnish Rheumatoid Arthritis Combination Therapy (FIN-RACo) trial, for instance, demonstrated that combination therapy is superior to monotherapy [12]. The Optimized Treatment Algorithm for Patients with Early Rheumatoid Arthritis (OPERA) study revealed that conventional treatment using MTX and frequent intra-articular glucocorticoid injections achieved very good results in early RA but that the addition of anti-TNF to this regimen yielded even better results, achieving remission in a remarkably high proportion of patients [13]. In the Rotterdam Early Arthritis Cohort (tREACH), researchers in the Netherlands studied the efficacy of triple DMARD therapy (MTX, sulfasalazine and hydroxychloroquine) with the use of glucocorticoids in early RA [14]. This study further confirmed that triple DMARD induction therapy is better than MTX monotherapy, and suggested that oral and intramuscular glucocorticoids were equally effective as bridging therapy. Another study showed that both triple therapy (sulfasalazine, hydroxychloroquine and MTX) and combination therapy (etanercept plus MTX) were effective in patients with RA who had active disease despite MTX therapy [15].

Taken together, these data show that different approaches can be used to induce disease remission in patients with early RA for whom MTX monotherapy fails. More work is clearly needed on the cost-effectiveness of conventional DMARD regimens compared to biological treatment. Considering cost-effectiveness, it is particularly important how biological therapy versus conventional combination treatment alters the work loss outcomes. In a recently published sub-analysis from the Swefot trial, working-age patients were studied. A similar reduction in the number of days with sick leave or disability pension was observed in both those patients receiving conventional DMARD combinations with MTX and those receiving anti-TNF [16].

An important development in the approach to early RA has been the demonstration, in several randomized trials, that frequent monitoring and a prespecified target yield better results - although with a more frequent usage of glucocorticoids and at higher costs - than the more common practice of three-monthly follow-up and adjusting treatments based on general impressions [17,18]. ‘Treat-to-target’ has become the accepted term for what is demonstrably a more successful approach. Using this approach and aiming for disease remission, a significant proportion of patients with RA will need biological treatment at some stage after optimal conventional DMARD treatment fails. Currently, 40% to 50% of patients with moderate to severe RA in the USA use biologics. Many of these patients will subsequently be using biological treatment for several years.

An important research question is whether the treatment paradigm could be changed in a cost-effective way by reversing the order of medicines that are used to treat RA. One such method would be to use an ‘induction-maintenance’ type of approach: to start the most effective agents, including biologics, very early in the course of the disease in the hope of achieving excellent results but also of being able to later withdraw these agents to minimize exposure to risks and achieve better cost-effectiveness. To what extent is such an approach supported by current data? First, biological treatment is more effective when initiated during the earliest stages of RA compared to later stages, an effect that has been shown for different mechanism of action, such as etanercept [19], tocilizumab [20], abatacept [21] and rituximab [22]. Based on these and other studies, it has been suggested that there is a therapeutic window of opportunity [23]. It is tempting to speculate that this clinical observation might be explained by the differences between early RA and late-stage RA in synovial tissue mass; progressive joint destruction, which may be lead to the release of proinflammatory crystals; and epigenetic changes in fibroblast-like synoviocytes, which could result in autonomous disease progression. The question is whether interfering with this process of autonomous disease progression with biological treatment during the earliest stage after the disease becomes clinically manifest could result in a disease that is easier to control over time with just conventional DMARDs. An early indication that this may be possible came from the small but randomized trial by Quinn *et al*. [24], where 10 patients were given MTX plus infliximab as first-line therapy for RA. After one year the biologic was discontinued and the excellent responses seen in most of the patients were maintained for an additional year of follow-up. The same initial treatment was used in the fourth arm of the Behandelings Strategiën (BeSt). Here, 120 patients received MTX plus infliximab for nine months and 87 achieved a good response; of those, 77 were able to maintain the response with MTX alone after the biologic had been stopped, again suggesting the effectiveness of induction-maintenance [25]. Very recently, these results were confirmed using initial treatment with MTX plus adalimumab in the Optimal Protocol for Methotrexate and Adalimumab Combination Therapy in Early Rheumatoid Arthritis (OPTIMA) study [26]. In this large randomized trial, 44% of patients achieved the low-disease-activity target at 24 to 26 weeks with the combination treatment (versus 24% with MTX plus placebo). These patients were then re-randomized to either continuation of the combination of both, or continuation with MTX plus placebo. After an additional year, 91% of patients in the continued combination group, and 81% in the MTX plus placebo continuation group had maintained low disease activity, demonstrating that for many patients the MTX maintenance therapy was effective. A formal cost-effectiveness analysis based on this trial has not yet been performed, but it seems reasonable to assume that induction-maintenance with biologics may not only be an effective way of treating the disease with rapid achievement of therapeutic targets in higher proportions of patients, but also a cost-effective way of using expensive agents for a limited period of time to bring the disease to a stable low-activity state (or to remission), which can then be sustained with simpler means. If the health-economic feasibility can be formally demonstrated, early start and stop of biologics may well become the new paradigm for treating early RA.

## Conclusions

Disease remission can be achieved in a significant proportion of patients with early RA by optimal use of conventional DMARDs. Biological DMARDs are indicated in patients with persistent disease activity, and are subsequently used for chronic treatment. We discuss the possibility of a novel treatment paradigm where biological DMARD treatment is used for a limited period of time during the so-called therapeutic window of opportunity to induce disease remission, followed by maintenance of remission with conventional DMARDs. Future research needs to address the question whether this may be a more cost-effective approach than the current treatment paradigm.

## Abbreviations

DMARDs: disease-modifying antirheumatic drugs; MTX: methotrexate; RA: rheumatoid arthritis; TNF: tumor necrosis factor.

## Competing interests

RvV has received research support from AbbVie, BMS, GSK, Pfizer, Roche and UCB; consultancy/honoraria from AbbVie, Biotest, BMS, GSK, Janssen, Lilly, Merck, Pfizer, Roche, UCB and Vertex. GN has received consulting/speaking fees from AbbVie, Berlin-Chemie Menarini, BMS, Egis, GSK, Pfizer, Roche, Takeda and UCB. PPT is currently an employee of GlaxoSmithKline.

## Authors’ contributions

RvV wrote the first draft of the manuscript, integrated the revisions by the other authors, and approved the final version. GN contributed to writing the manuscript and approved the final version. PPT contributed to writing the manuscript and approved the final version.
